# Amyloid Beta Inhibits Olfactory Bulb Activity and the Ability to Smell

**DOI:** 10.1371/journal.pone.0075745

**Published:** 2013-09-26

**Authors:** Reynaldo Alvarado-Martínez, Karla Salgado-Puga, Fernando Peña-Ortega

**Affiliations:** Departamento de Neurobiología del Desarrollo y Neurofisiología, Instituto de Neurobiología, Universidad Nacional Autónoma de México, UNAM, Campus Juriquilla, Querétaro, México; Université Lyon, France

## Abstract

Early olfactory dysfunction has been consistently reported in both Alzheimer’s disease (AD) and in transgenic mice that reproduce some features of this disease. In AD transgenic mice, alteration in olfaction has been associated with increased levels of soluble amyloid beta protein (Aβ) as well as with alterations in the oscillatory network activity recorded in the olfactory bulb (OB) and in the piriform cortex. However, since AD is a multifactorial disease and transgenic mice suffer a variety of adaptive changes, it is still unknown if soluble Aβ, by itself, is responsible for OB dysfunction both at electrophysiological and behavioral levels. Thus, here we tested whether or not Aβ directly affects OB network activity *in vitro* in slices obtained from mice and rats and if it affects olfactory ability in these rodents. Our results show that Aβ decreases, in a concentration- and time-dependent manner, the network activity of OB slices at clinically relevant concentrations (low nM) and in a reversible manner. Moreover, we found that intrabulbar injection of Aβ decreases the olfactory ability of rodents two weeks after application, an effect that is not related to alterations in motor performance or motivation to seek food and that correlates with the presence of Aβ deposits. Our results indicate that Aβ disrupts, at clinically relevant concentrations, the network activity of the OB *in vitro* and can trigger a disruption in olfaction. These findings open the possibility of exploring the cellular mechanisms involved in early pathological AD as an approach to reduce or halt its progress.

## Introduction

In recent decades, it has been demonstrated that at very early stages, Alzheimer’s patients find it difficult to detect, discriminate, and identify odors [1-6]. Such dysfunction has been correlated with early pathological findings such as sparse accumulation of amyloid beta protein (Aβ) in the olfactory bulb (OB) [7-11]. Similar observations of reduced olfaction [12-20] as well as early histopathological changes in the OB [15,20-24] have been reported in transgenic mice that overexpress proteins associated with familial AD. More recently, a strong correlation has been established between the accumulation of soluble forms of Aβ in the OB and very early olfactory dysfunction in both AD patients and transgenic mice [15]. A similar correlation was found in young human subjects exposed to ambient pollution, such as that found in Mexico City, who exhibit reduced olfactory abilities along with Aβ accumulation in the OB [25,26]. Accordingly, experimental strategies that reduced the amount of Aβ also reduced the olfactory dysfunction observed in AD transgenic mice [17,19]. Taken together, this evidence suggests a causal relationship between Aβ and olfactory dysfunction. However, the effect of Aβ applied directly onto the different centers involved in the olfactory neural pathway has not been tested.

The OB is the first relay center and integrator in the olfactory information-processing pathway [27,28]. Starting with the pioneering studies performed by Adrian (1950) more than 7 decades ago, it has been shown that the OB generates a variety of network population activities that seem to be involved in the detection and signal processing of olfactory information [29-40]. For example, it was shown that the magnitude of the fast oscillatory activity generated by the OB is proportional to the olfactory task demand or performance [30,38-40]. Moreover, alterations in several of the cellular mechanisms involved in generating patterns of population activity by the OB, such as the dendrodendritic interaction between mitral and granular cells, affect the ability of the OB to represent and process olfactory information [30,32-35,41]. In addition, several experimental manipulations that either increase or decrease OB network activity improve or reduce, respectively, the rodents’ olfactory function [42-44].

Due to the facts that soluble Aβ is able to disrupt network activity in several circuits throughout the brain [45-51] and that such disruption is associated with deficits in behaviors regulated or commanded by such networks (i.e., learning and memory in the hippocampus [46,50]), it is likely that Aβ disrupts OB network activity and therefore affects olfactory function [15]. We tested this hypothesis by applying clinically relevant concentrations of Aβ directly onto OB slices of mice and rats [52-55], while recording their population activity in the granule cell layer and by testing olfactory performance in rodents injected with Aβ. Our results show that Aβ decreases, in a concentration- and time-dependent manner, the network activity of OB slices at clinically relevant concentrations (low nM) and in a reversible manner. Moreover, we found that intrabulbar injection of Aβ decreases the olfactory ability of rodents two weeks after application, an effect that persists for at least two more weeks, correlating with the presence of Aβ deposits. These findings indicate that Aβ plays a major role in the alterations observed in OB function and olfactory performance in both AD transgenic mice and patients.

## Materials and Methods

### Ethics Statement

Experiments were approved by the Committee on Bioethics of the Instituto de Neurobiología, UNAM and were carried out according to the Norma Oficial Mexicana de la Secretaría de Agricultura (SAGARPA NOM-062-ZOO-1999), which complies with the guidelines in the Institutional Animal Care and Use Committee Guidebook (NIH publication 80-23, Bethesda, MD, USA, 1996).

### Subjects

Wistar rats (8 weeks old) as well as CD-1 mice of 3, 6, and 8 weeks of age were obtained from the animal facility of the Institute of Neurobiology, UNAM, where they were kept under a normal 12-h light: 12-h dark cycle (lights on at 7:00 a.m.) with free access to food and water. Three triple transgenic mice [56] were obtained from the same source and used as positive controls for thiazine red staining.

### Aβ Preparation

Aβ was obtained from Bachem (Heidelberg, Germany) and oligomerized using a standard protocol described earlier [52,57]. Briefly, 1,1,1,3,3,3-hexafluoro-2-propanol (HFIP) was added to solid amyloid beta_1-42_ at a final peptide concentration of 1 mM and incubated for 60 min at room temperature. HFIP was allowed to evaporate overnight, and a 5 mM solution was prepared by adding DMSO. This solution was diluted with F12 medium to reach a final concentration of 100 µM and then incubated at 5°C for 24 h. Then the solution was centrifuged at 14,000 × *g* for 10 min in the cold. The supernatant, containing the amyloid beta oligomers, was collected and used for the experiments. Characterization of this solution by electrophoresis showed the presence of both amyloid beta monomers and oligomers [52].

## In Vitro Experiments

### Olfactory Bulb Slice Preparation

To obtain OB slices, mice and rats were anesthetized with sodium pentobarbital (50 mg/kg, intraperitoneally (i.p.)) and perfused transcardially with cold protective saline containing (in mM): 250 glycerol, 3 KCl, 36 NaHCO_3_, 5 KH_2_PO_4_, 10 glucose, 0.7 CaCl_2_, and 2 MgSO_4_, pH 7.4, bubbled with carbogen (95% O_2_ and 5% CO_2_). Then, animals were decapitated; the olfactory bulbs were removed and dissected in ice-cold artificial cerebrospinal fluid (aCSF) containing (in mM): 119 NaCl, 3 KCl, 1.5 CaCl_2_, 1 MgCl_2_, 25 NaHCO_3_, and 30 D-glucose, pH 7.4, and bubbled with carbogen (95% O_2_ and 5% CO_2_). One olfactory bulb was glued to an agar block, and 400 µm thick parasagittal slices were cut with a vibratome (Vibratome, St. Louis, MO, U.S.A.). Slices were allowed to recover in aCSF at room temperature for at least 60 min before any further experimental manipulation.

### Population Recordings

For extracellular field recordings, the OB slices were transferred to a submerged recording chamber continuously perfused at 17-20 ml/min with oxygenated aCSF maintained at 30-32 °C. The field recordings were obtained with suction electrodes filled with aCSF and positioned on the granule cell layer using a stereoscopic microscope and a micromanipulator. The signal was amplified and filtered (highpass, 0.5 Hz; lowpass, 1.5 KHz) with a wide-band AC amplifier (Grass Instruments, Quincy, MA, U.S.A.). OB activity was recorded for 15-20 min to obtain the control recording in each slice before adding Aβ to the bath at three concentrations (3 nM, 10 nM, and 30 nM) to test its effect on network activity for 1 h. Finally, 1 mM lidocaine was added to the bath to block any neural activity, as a control for the viability of the slice. In some experiments, after testing the effect of 30 nM Aβ for 1 h, Aβ was washed out of the bath for at least 1 h to test for the reversibility of the Aβ effect. In another set of slices, we evaluated the effect of bath application of 30 nM inverse amyloid beta_42-1_ on OB activity network. All recordings were digitized at 3-9 KHz and stored on a personal computer with an acquisition system from National Instruments (Austin, TX, U.S.A.) using custom-made software designed in the LabView environment.

## In Vivo Experiments

### Surgical Procedure

Adult male Wistar rats (300-350 g at the time of surgery) were acclimated to the laboratory *vivarium* for at least one week before surgery. There, animals were housed individually in a temperature-controlled (24°C) room and maintained on 12-h/12-h light/dark cycle (lights on at 7:00 a.m.). Food and water were provided *ad libitum* throughout the experiment. On the day of Aβ or vehicle (F12 medium) microinjection into the olfactory bulbs, the animals were anesthetized with sodium pentobarbital (50 mg/kg i.p.), given an extra application of atropine sulfate (1 mg/kg i.p.), and secured in a stereotaxic frame (Stoelting Co., IL) as previously described [45]. The co-ordinates used were: AP 9.4 from bregma, L ± 1.3 from midline, and V -2.5 from dura [58]. A 1-µl volume containing 100 pmoles of Aβ was bilaterally injected at a rate of 0.2 µl/min (200 pmoles of Aβ per animal); 5 min later, the needle was withdrawn and the skin sutured.

### Behavioral Measurements

Seven days after surgery, animals were tested for olfactory function [20,59-61]. The fed rats were placed in a cage (24 × 20 × 45 cm) with clean sawdust covering the floor. In each test, a 50-mg piece of chocolate (TRIKI-TRAKES®) was randomly placed at one of the four corners, hidden under the sawdust. The time the animal took to reach the chocolate was recorded. The maximum test time allowed was 600 s. For statistical analysis only the rats in which the perfusion injectors were located in the OB region were included. Based on previous reports, the test does not require food or water deprivation [59-62], although it can be performed after food-deprivation [20,61,62] with no evident differences between the two conditions [62]. In all cases, the inability to find (see pp 15-16 for explanation) the hidden food is interpreted as an alteration in the main olfactory bulb function [20,59-61]. Alternatively, to exclude both motor deficits and motivational failure, we repeated the same “olfactory test” with a food pellet (normal chow) in a visible position in front of the rat [62]. Moreover, to rule out any motivational contribution that could be interfering with the olfactory assessment, the hidden food test was performed in other groups of animals injected either with vehicle or with the same amount of Aβ, while food-deprived during the third week post-injection or while fed during the fourth week post-injection. The same animals were also tested for food consumption [63] by measuring the amount of food eaten in 30 min and 120 min. All rats were placed individually in clean sawdust cages with 50 g of chow. We quantified the amount of food eaten by the animals at the end of the third post-injection week in 24-h food-deprived animals and repeated the quantification at the end of the fourth post-injection week in the same animals maintained with food *ad libitum*. We also measured food consumption during one 24-h period, once a week (which did not differ between groups, data not shown), and quantified weight gain throughout the four weeks post-injection to test for changes in feeding behavior that would alter our olfactory test observations [63].

### Histological Evaluation

Histological evaluation of the injection sites [64-66], as well as thiazine red staining [67-69] were performed as follows: Animals were anesthetized with sodium pentobarbital (50 mg/kg i.p.), transcardially perfused with 250 ml of 0.9% NaCl followed by 250 ml of 4% paraformaldehyde in 0.1 M phosphate buffer (pH 7.4), and brains were removed and prepared for the histological procedure, as described previously [64,65]. Sagittal sections (50 µm thick) were stained either with toluidine-blue [66] to verify the injection sites or with thiazine red to look for Aβ deposits [67-69].

### Statistical Analysis

The recordings obtained were analyzed off-line by performing classical power spectrum analysis [70-72]. Segments of 20 s every 5 min were analyzed using a Rapid Fourier Transform Algorithm with a Hamming window, in Clampfit (Molecular Devices). The power spectra, from 1 to 50 Hz, were averaged and plotted for the last two segments of each experimental condition. We also evaluated the power spectra by measuring different frequency bands such as theta (2-12 Hz), beta (13-35 Hz) and gamma (35-50 Hz). The spectra were integrated and normalized to the control, i.e., the control power spectrum was integrated, and this value was arbitrarily set as 100%. Finally, peak frequency was determined as the frequency with the maximal power in each spectrum.

We used the Prism and Sigma Plot programs to prepare the graphs and the descriptive and inferential statistics. A Kruskal-Wallis analysis was used for statistical comparisons between experimental conditions. Upon finding a significant difference, we performed the Dunn’s test, and values of p < 0.05 were considered significant. In all cases reported in the text, the data are reported as means ± S.E.M. To compare the effects of the three Aβ concentrations tested at the three different ages, we used the Friedman test followed by Dunn’s test. Additionally, to evaluate the effects of Aβ compared with its own control the Wilcoxon test was used. Since the olfactory capacity test was truncated at 600 s, for these experiments only, the data is represented in the figures as the median and the interquartile range.

## Results

### Aβ reduces OB network activity in a reversible and specific manner

**Figure 1:**
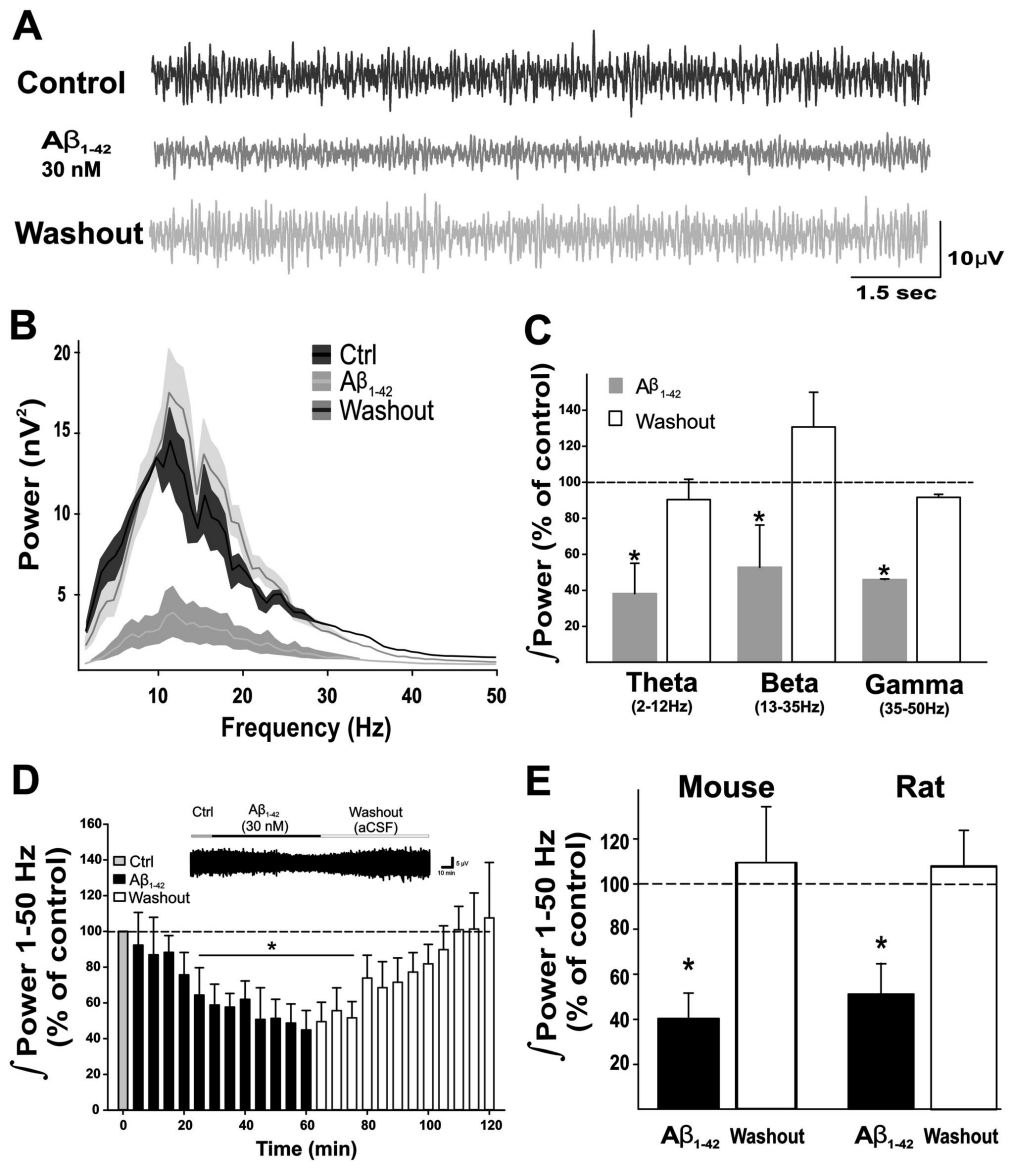
Amyloid beta (Aβ) inhibits spontaneous network activity in the olfactory bulb (OB) of mice and rats. A, Representative recordings of the OB spontaneous network activity in slices, filtered at highpass = 0.5 Hz and lowpass = 1.5 KHz, obtained from 8-week-old mice in control conditions (upper trace), after 60 min of Aβ exposure (middle trace), and after 30 min of washout (lower trace). B, Averaged power spectra (± standard error; shaded area) of the slices under the three experimental conditions shown in A. Note that OB spontaneous network activity includes a broad variety of frequencies and that Aβ application produces a generalized and reversible reduction of the power without preferentially affecting any frequency range. C, Quantification of the power of OB network activity (as % of control) for different frequency bands (theta, beta, and gamma) for the experimental conditions represented in A. Note that Aβ reversibly reduces the power of the OB network activity to the same degree for all frequencies. D, Time-course of Aβ-induced inhibition of OB network activity, as % of control, over a frequency range of 1 to 50 Hz, and its washout. E, Comparison of Aβ-induced inhibition of OB network activity and its washout recorded in slices obtained from mice and rats. Note that the phenomenon is identical in slices obtained from both species. * Denotes a significant difference (p < 0.05) relative to the control (n = 7).

The OB network activity recorded in the granule cell layer of slices obtained from 8-week-old mice shows low-voltage neuronal activity (Figure 1A, top trace) that contains a broad range of frequency components (Figure 1B,C) with a peak frequency (frequency with maximal power) of 13.3 ± 2.5 Hz (n = 7). The activity described here has very similar characteristics to that reported in previous studies *in vitro* [35,73-76] and resembles that recorded *in vivo* [38,77,78]. Continuous bath application of 30 nM Aβ for 1 h induces a significant inhibition of OB network activity (Figure 1A, middle trace) with proportional reductions in the activity at the frequencies corresponding to theta (to 38.2 ± 15.8% of control, Figure 1C), beta (to 52.8 ± 23.4% of control, Figure 1C), and gamma bands (to 45.9 ± 0.36% of control, Figure 1C). Consequently, Aβ application does not affect peak frequency of the OB network activity (10.7 ± 3.8 Hz, Figure 1B). Quantification of the OB network activity over the whole range between 1 and 50 Hz shows that after 1 h of continuous Aβ application, such activity is reduced to 40.1 ± 11.3% of control (Figure 1D,E). The time-course of the Aβ-induced inhibition of OB network activity shows that the reduction can already be observed after 15 min of continuous Aβ application (to 75.7 ± 12.5% of control, Figure 1D) and that it reaches significance after 25 min of continuous Aβ application (to 58.8 ± 11.7% of control, Figure 1D). The Aβ-induced inhibition of OB network activity described for slices obtained from mice (Figure 1E) is identical to that observed in slices obtained from rats of the same age (to 51.3 ± 13.7% of control, Figure 1E). The use of this *in vitro* approach allows us to demonstrate that, after washout of Aβ from the bath the OB network activity recovers to control levels in slices obtained from both species (109.3 ± 24.7% of control in mice and 108.0 ± 16.2% of control in rats; Figure 1C-E).

**Figure 2:**
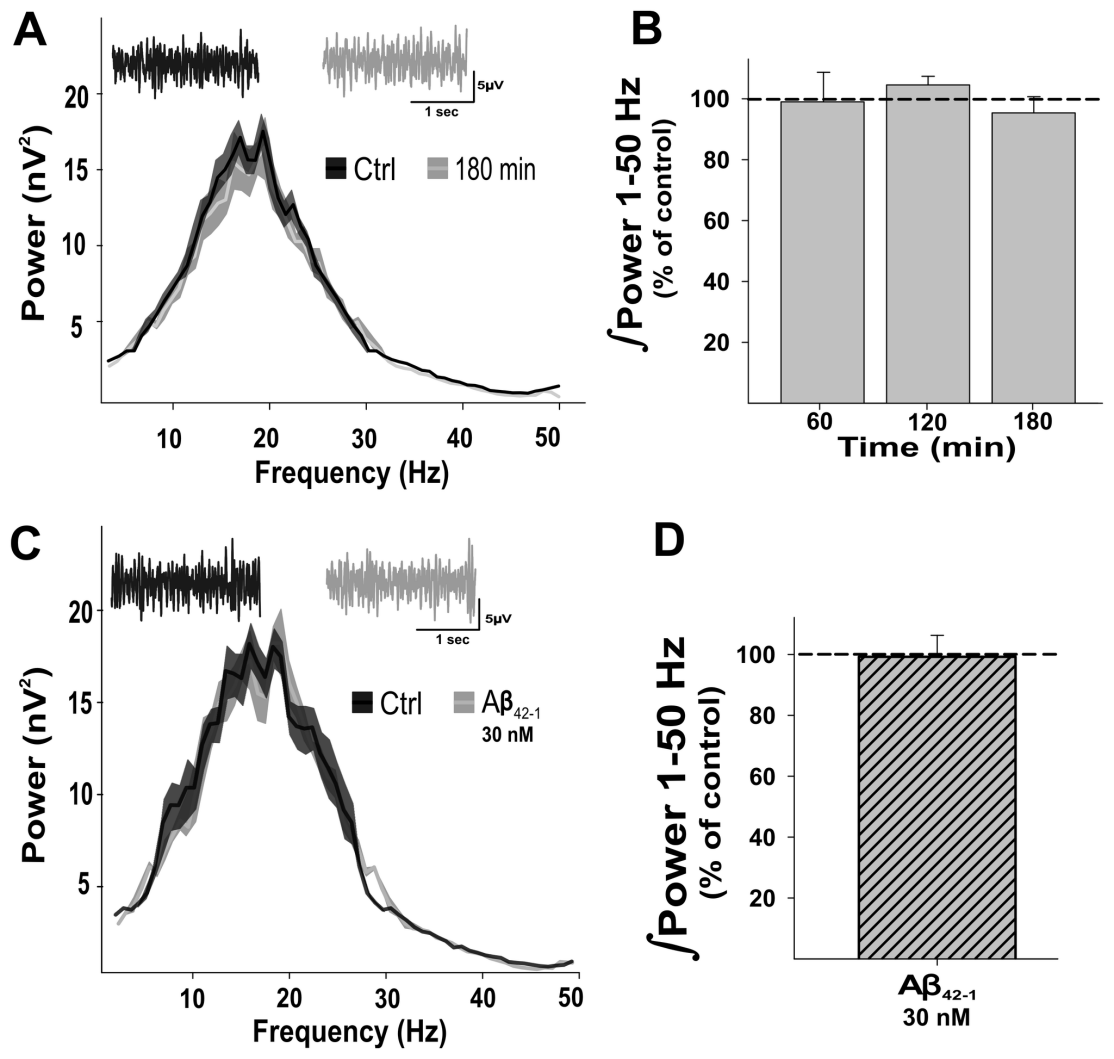
OB network activity is stable through time and is not affected by the inverse Aβ sequence. A, Averaged power spectra (± standard error) of the slices in control conditions (dark gray) and after 3 h of continuous recording under the same control conditions (light gray). The insets shows representative recordings, filtered at highpass = 0.5 Hz and lowpass = 1.5 KHz for both conditions using the same color code. B, Quantification of the power of OB network activity (as % of control) over a frequency range of 1 to 50 Hz at three different time points. Note that OB network activity remains unaltered for at least 3 h of recording. C, Averaged power spectra (± standard error) of the slices in control conditions (dark gray) and after 1 h of continuous application of the Aβ inverse sequence (42-1, light gray). The insets show representative recordings for both conditions (using the same color code). D, Quantification of the power of OB network activity (as % of control) over a frequency range of 1 to 50 Hz 1 h after application of the inverse Aβ sequence, showing no effect of the peptide.

Two control experiments indicate that these effects are due to specific actions of Aβ on the OB network. First, we demonstrated that long-lasting recording of OB slices does not affect network activity. The power of OB network activity remained unaltered after 60 min (99.5 ± 8.4% of control), 120 min (104.4 ± 2.3%), or 180 min (97.1 ± 2.9% control) of continuous recording (Figure 2A). Similarly, the peak frequency also remained unaltered (13.2 ± 0.8 Hz; 12.8 ± 1.4 Hz; 13.8 ± 1.6 Hz, after 60, 120, and 180 min, respectively). Moreover, we demonstrated that the application of the Aβ-inverse sequence (,42) does not affect OB network activity (Figure 2C,D). After 1 h of continuous bath application of the Aβ-inverse sequence, the power of OB network activity (99.6 ± 5.4% of control, n = 6) (Figure 2D) as well as its peak frequency (16.6 ± 2.8 Hz) remained unaltered (Figure 2C).

### The sensitivity of OB network activity to Aβ increases with age

**Figure 3:**
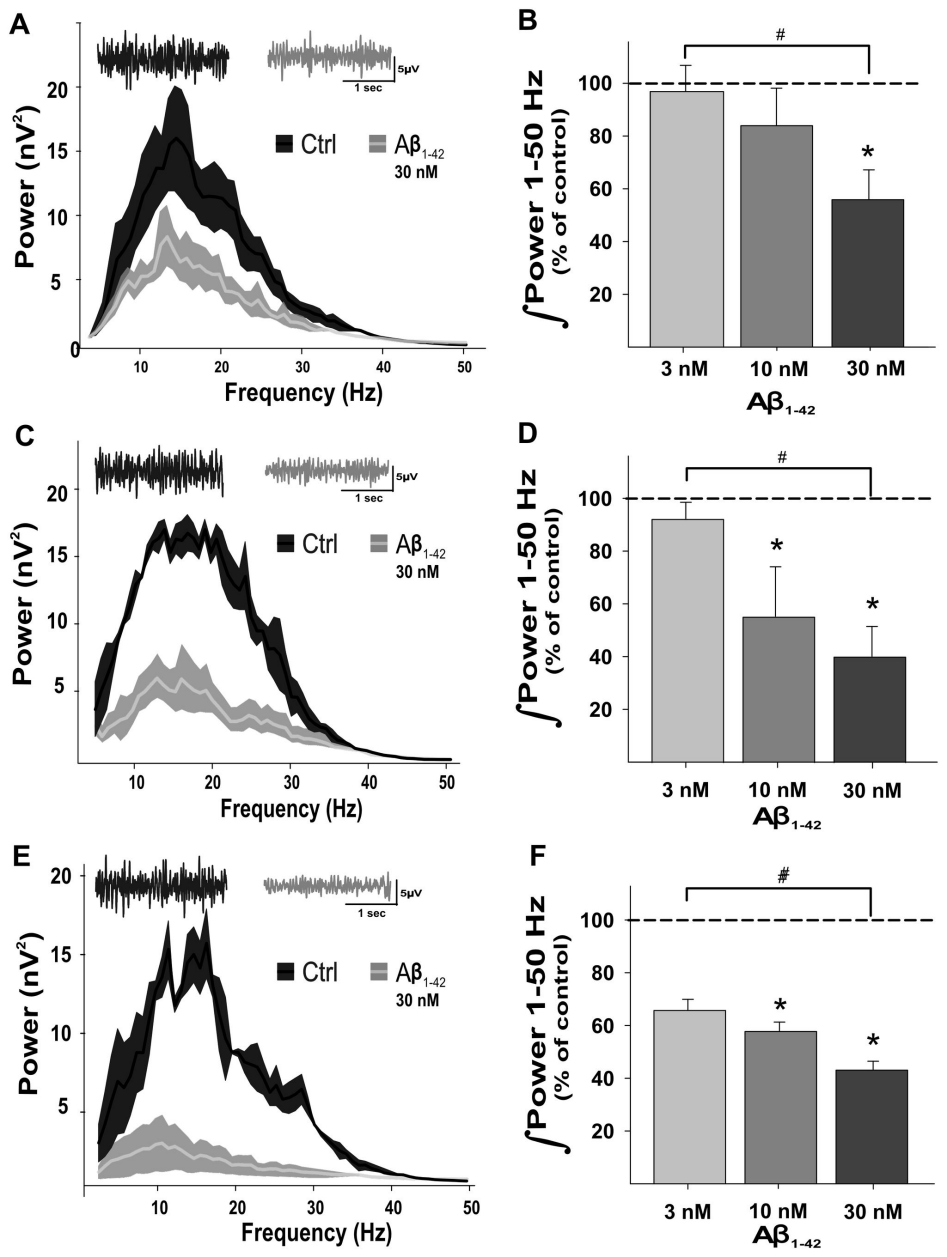
Aβ-induced inhibition of OB network activity is age and concentration dependent. A, Averaged power spectra (± standard error) of the slices obtained from 3-week-old animals in control conditions (dark gray) and after 1 h of continuous application of 30 nM Aβ (light gray). The inset shows representative recordings, filtered at highpass = 0.5 Hz and lowpass = 1.5 KHz, for both conditions using the same color code. B, Quantification of the power of OB network activity (as % of control) over a frequency range of 1 to 50 Hz after the application of different concentrations of Aβ to slices obtained from 3-week-old animals. Note that Aβ reduces OB network activity only at the highest concentration tested. C and D, Same as A and B except the slices were obtained from 6-week-old animals. Note that Aβ reduces OB network activity at the two highest concentrations tested. D and F, Same as A and B except the slices were obtained from 8-week-old animals. Note that Aβ reduces OB network activity at the two highest concentrations tested and that 3 nM Aβ also tends to reduce the activity. * Denotes a significant difference relative to control, and # denotes a significant difference between conditions (p < 0.05).

The effects of Aβ seem to be time dependent [52]; therefore, we tested the effects of three clinically relevant concentrations of Aβ (3, 10, and 30 nM) on slices obtained from mice at three different ages (3, 6, and 8 weeks old). The OB network activity recorded in the granule cell layer of slices obtained from 3-week-old mice shows low-voltage neuronal activity (Figure 3A, insets) that contains a broad range of frequency components (Figure 3A) and a peak frequency of 12.4 ± 0.6 Hz (n = 7). Continuous bath application of 3 nM Aβ for 1 h does not affect either the power of OB network activity (96.7 ± 9.5% of control; Figure 3B) or its peak frequency (13.4 ± 0.7 Hz). Subsequent increase of the Aβ bath concentration to 10 nM for 1 h does not affect OB network activity either (Figure 3B), since the power of the activity remains at 80.4 ± 13.7% of control, and the peak frequency did not change (12.8 ± 1.5 Hz). In contrast, subsequent increase of the Aβ bath concentration to 30 nM for 1 h induces a significant reduction in OB network activity (Figure 3A,B) to 53.2 ± 10.7% of control. The inhibition of OB network activity induced by Aβ affects all frequency components proportionally (Figure 3A) and does not affect peak frequency (12.0 ± 0.4 Hz).

The OB network activity recorded in the granule cell layer of slices obtained from 6-week-old mice showed low-voltage neuronal activity (Figure 3C, insets) that contained a broad range of frequency components (Figure 3C) and a peak frequency of 13.3 ± 2.6 Hz (n = 7). This activity was slightly more sensitive to the presence of Aβ than the activity recorded in slices obtained from younger animals. Continuous bath application of 3 nM Aβ for 1 h affected neither the power of OB network activity (93.2 ± 5.6% of control; Figure 3D) nor its peak frequency (10.0 ± 4.7 Hz). In contrast to slices obtained from younger animals, subsequent increase of Aβ bath concentration to 10 nM for 1 h induced a significant reduction in OB network activity (Figure 3D) to 54.2 ± 19.4% of control, inhibiting all frequency components proportionally but without altering peak frequency (7.8 ± 1.7 Hz). A subsequent increase of Aβ bath concentration to 30 nM for 1 h induced no further reduction in OB network activity (44.1 ± 11.8% of control; Figure 3C, D); however, the power of OB network activity in the presence of 30 nM Aβ was significantly lower than the power in the presence of 3 nM Aβ, but this was not the case for 10 nM Aβ (Figure 3D). The inhibition of OB network activity induced by Aβ 30 nM affected all frequency components proportionally but did not affect peak frequency (8.7 ± 1.73 Hz, Figure 3C).

The OB network activity recorded in the granule cell layer of slices obtained from 8-week-old mice shows low-voltage neuronal activity (Figure 3E, insets) that contains a broad range of frequency components (Figure 3E) with a peak frequency of 16.4 ± 1.7 Hz (n = 7). Such activity tends to be more sensitive to the presence of Aβ than the activity recorded in slices obtained from younger animals. Bath application of 3 nM Aβ induces reduction in OB network activity (Figure 3F) to 65.2 ± 4.9% of control; this reduction did not reach statistical significance when tested by Dunn’s test (after a Friedman test), but it was significantly different from the control when evaluated by the Wilcoxon test (p < 0.05). This inhibition affected all frequency components proportionally and did not affect peak frequency (19.0 ± 2.0 Hz). A subsequent increase of Aβ bath concentration to 10 nM for 1 h induced a significant reduction in OB network activity (Figure 3F) to 54.6 ± 5.2% of control, again inhibiting all frequency components proportionally without affecting peak frequency (15.7 ± 1.9 Hz). Increasing the Aβ bath concentration to 30 nM for 1 h induced a further reduction in OB network activity to 46.8 ± 4.1% of control (Figure 3E, F), again with proportional effects on all frequency components (Figure 3E) but no effect on peak frequency (12.6 ± 0.9 Hz). In the presence of 30 nM Aβ, but not of 10 nM Aβ, the power of the OB network activity was significantly lower than the power in the presence of 3 nM Aβ (Figure 3E). The effects of the different Aβ concentrations on slices obtained at different times (3, 6, and 8 weeks old) showed no significant differences when compared using the Friedman test followed by Dunn’s post test, considering both time and concentration as variables.

### A single intrabulbar injection of Aβ induces a time-dependent loss of smell

**Figure 4:**
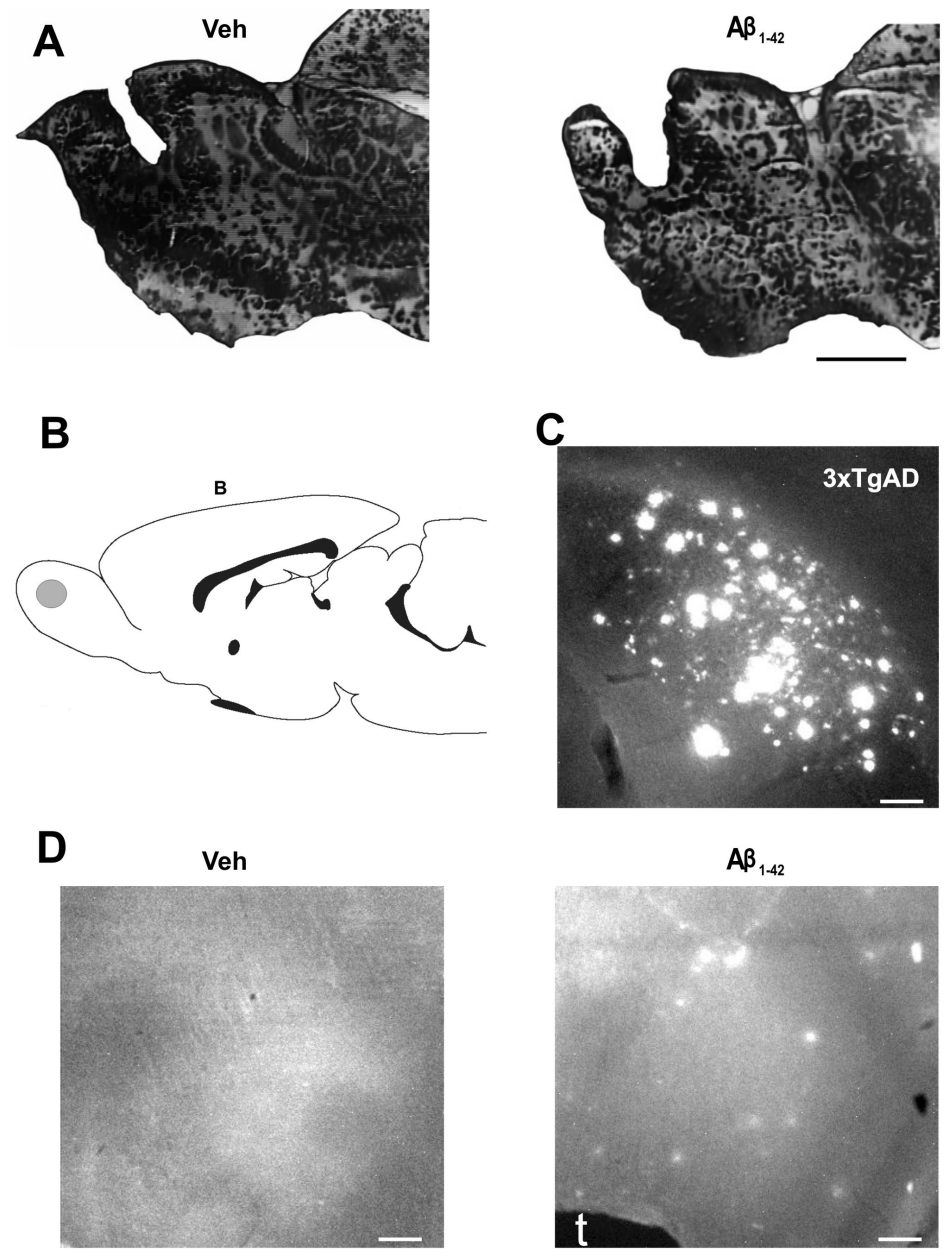
Intrabulbar Aβ application induces permanent deposits. A, Photomicrographs of histological sections stained with toluidine-blue taken from both vehicle (left) and Aβ-injected (right) animals showing the lesion induced by the microinjector. Note that the microinjector reaches the granule cell layer. B, Schematic representation (adapted from Paxinos and Watson [58]) of the microinjector tip locations, represented as a gray circles, in both bulbi of all animals. B denotes the position of Bregma. C, Photomicrograph illustrating thiazine red positive plaques stained in the *subiculum* of a 19-month-old triple transgenic (3xTgAD) mouse. D, Photomicrographs of histological sections, stained with thiazine red, from both vehicle (left) and Aβ-injected (right) animals. Note that Aβ-treated animals exhibit thiazine red deposits, whereas vehicle-treated animals do not. The t denotes the track left by the microinjector. Scale-bars denote 1 mm for A, and 200 µm for C and for the micrographs in D.

**Figure 5:**
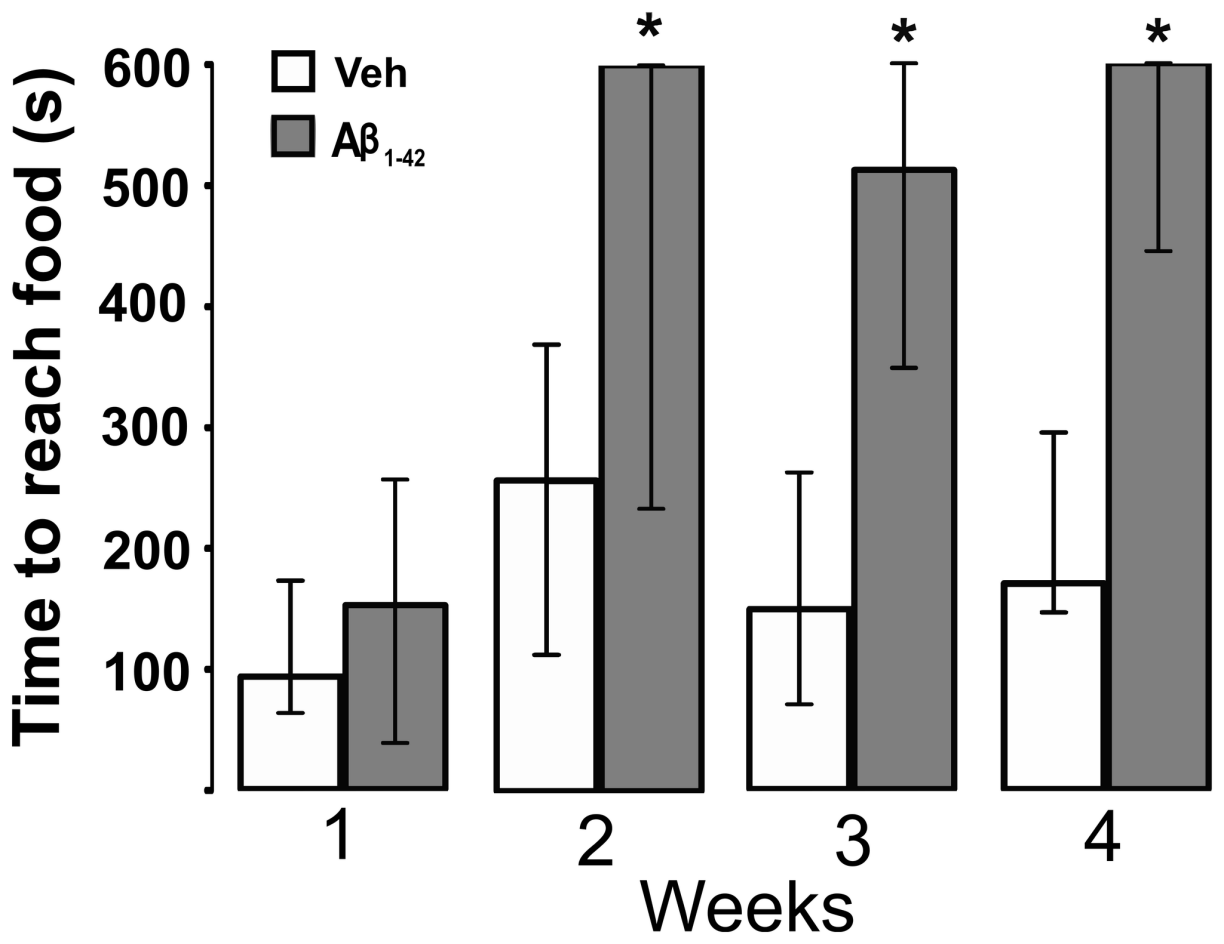
A single intrabulbar Aβ application impairs the ability to smell. The graph shows the time needed for control animals (injected with vehicle; white bars) and animals with intrabulbar injection of Aβ (gray bars) to reach a hidden piece of chocolate (50 mg). The horizontal axis indicates when the test was made, expressed as the number of weeks after surgery. The maximal time allowed for the search was 600 s. Note that the animals lose the ability to smell, and the loss increases with time after the Aβ injection. For these experiments the data are represented as the median and the interquartile range. * Denotes a significant difference relative to control (vehicle; p < 0.05).

**Figure 6:**
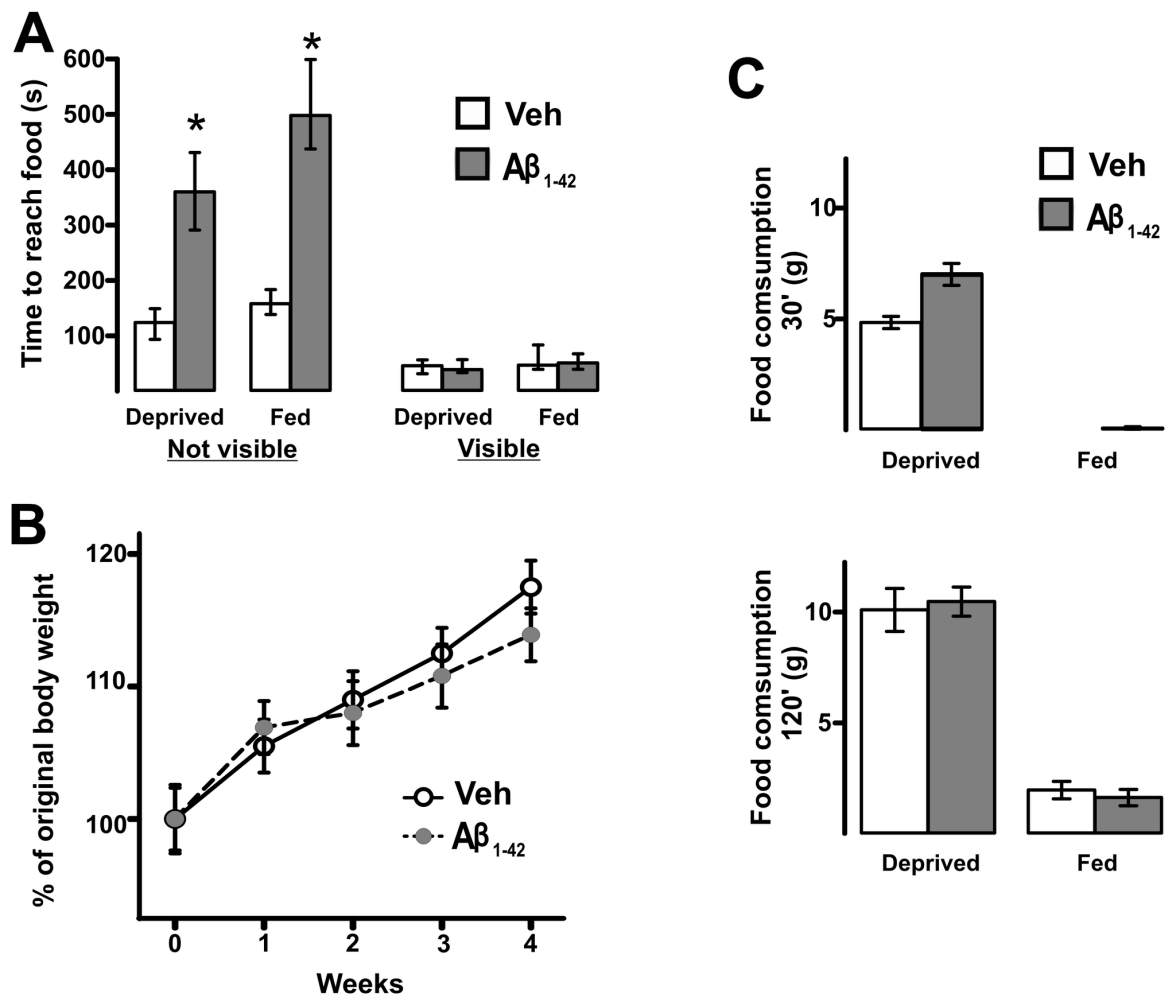
Aβ-induced olfactory disruption is not associated with alterations in motivation to seek food or motor performance. A, Quantification of time needed to reach food for control animals (injected with vehicle; white bars) and animals with intrabulbar injection of Aβ (gray bars) in two conditions: Food-deprived and Fed. Additionally, animals were tested while food was either hidden (a chocolate piece) or in plain sight (normal chow). Note that the animals injected with Aβ exhibit a deficit in their ability to find the hidden food, regardless of their feeding status. In contrast, no difference was found for any experimental group when the food was visible. Note that just for these experiments the data are represented as the median and the interquartile range. B, Time-course of body weight increase, quantified as % of the original weight (on the day of surgery), for animals injected with vehicle and animals with intrabulbar injection of Aβ. C, Food intake of control animals injected with vehicle (white bars) and animals with intrabulbar injection of Aβ (gray bars) tested at two time points (30 min upper graph and 120 min, lower graph). * Denotes a significant difference relative to control (vehicle; p < 0.05).

To assess the behavioral consequence of Aβ-induced neural network alterations, we injected Aβ_1-42_ into the olfactory bulb (100 picomoles/side, n = 18; Figure 4) and tested olfactory function by the ability of the animals to find a hidden piece of chocolate [59-62] while either normally fed or food deprived. We verified that the tip of the microinjector reached the granule cell layer as a criterion to include the data obtained from a given animal in the behavioral analysis (Figure 4A,B). Moreover, we found that intrabulbar injection of Aβ induced thiazine red positive deposits in the olfactory bulb that can be observed four weeks after injection (Figure 4D) but that are absent in control animals (Figure 4D). As a positive control, we observed abundant thiazine red staining in the brain of the triple transgenic mice (Figure 4C), which strongly suggests that thiazine red is staining Aβ deposits [56,67-69]. In these animals, we found that intrabulbar injection of Aβ induced a time-dependent loss of smell. One week after Aβ application (n = 9), the ability of the animals to the hidden chocolate remained unaltered compared with animals injected with vehicle (medium F12; n = 10; 155.7 ± 43.5 vs. 120.1 ± 24.2 s, respectively; Figure 5). However, only two weeks after Aβ application, the ability of the animals to find the hidden chocolate was seriously compromised (431.9 ± 69.0 s). At this time, 5 out of 9 animals failed to locate the piece of chocolate, and the average latency to reach the hidden food was significantly longer compared both to the same animals one week after Aβ application and to the control animals two weeks after vehicle injection (Figure 5). A similar result was found three and four weeks after Aβ application when the ability of the animals to find the hidden chocolate remained seriously impaired (450.9 ± 63.0 and 520.0 ± 45.9 s, respectively); at these times, 4 and 5 out of 9 animals, respectively, failed to reach the piece of chocolate. In both cases, the average latency to reach the hidden food was significantly longer than that of the same animals one week after Aβ application, and it was also longer than that of the control animals three and four weeks after vehicle injection (Figure 5). In another set of animals (n = 9 for each group), we performed control measurements to test whether or not the differences in the time to reach the hidden chocolate was due to changes in motivation to seek food [61-63]. First, we observed that the differences in the ability to find the hidden chocolate between control animals and those injected with Aβ was the same with or without a motivation imposed by hunger (Deprived for 24 h vs. normally fed; Figure 6A). We also excluded the possibility that intrabulbar injection of Aβ induces motor deficits or motivational failure by finding no difference between control and experimental groups in the time required to reach a food pellet that was visible to the animals (Figure 6A [61]). Moreover, there was no difference between control animals injected with vehicle and animals with intrabulbar injection of Aβ when measuring parameters related to feeding, such as weight gain (Figure 6B) or food intake (Figure 6C [63]).

## Discussion

Here, we found that soluble Aβ oligomers reversibly alter the activity of the OB in slices obtained from mice and rats and that their injection triggers a delayed loss of the ability to smell that might be related to the presence of Aβ deposits but not to a change in the motivation to seek food. We also found that Aβ-induced inhibition of OB network activity is time and concentration dependent, and occurs at concentrations similar to those found in AD patients [52-55]. Since the amyloid hypothesis indicates that the soluble Aβ oligomers are responsible for neural dysfunction [55,79] and because different varieties of neural functions correlate with the generation of certain types of spontaneous oscillatory activity [29-33,37,51], our results might provide the cellular basis of the early olfactory dysfunction observed both in AD patients and AD transgenic mice. Moreover, our findings confirm previous evidence that experimental manipulations alter the generation of synchronized activity by the OB and also produce olfactory deficits [42-44]. Importantly, as mentioned, Aβ induces OB network dysfunction at concentrations (low nanomolar) very close to those found in the brain of AD patients [53,54], and which closely correlate with the concentrations that produce oscillatory alterations in these patients [80,81].

The spontaneous OB network activity of slices recorded in our experimental conditions is very similar to that reported by other groups using a similar experimental approach [35,73-76]. Since this activity maintains the features of that generated by the OB *in vivo* [38,77,78], the OB slices can be used to test the effects of Aβ in controlled physiological conditions and at clinically relevant concentrations as well as the reversibility of these effects [52].

Another major finding of our study is that Aβ-induced OB network dysfunction is time dependent. The olfactory bulb is a network highly sensitive to AD pathology [1-10,15,20,23], and it is one of the first brain structures to accumulate Aβ; it shows functional deterioration even before other neural networks involved in cognitive performance, such as the hippocampus and the cholinergic nuclei [15,82]. As the brain becomes older, it becomes more sensitive to several insults. We found that the OB, like the hippocampus, becomes more sensitive to the effects of Aβ with age [52]. In fact, this increase in sensitivity to the effects of Aβ is more pronounced in the OB. For instance, we found that the network activity generated by the OB can be reduced by the application of 30 nM Aβ in slices taken from 3-week-old animals (Figure 3), whereas it was necessary to apply 100 nM Aβ to induce a similar reduction in network activity of hippocampal slices taken from 2-week-old animals [52]. Aging has been associated with loss of synaptic contacts [83,84], silencing of synapses [83-85], or a decrease of postsynaptic responsiveness [83,84,86,87] and synaptic dynamics [68, for review see 87], which is reflected in deficits in the generation of certain spontaneous oscillatory activities [52,88]. It remains to be determined which of these phenomena is responsible for the age dependency of Aβ-induced OB network dysfunction.

As observed for the hippocampus [52], we demonstrated that Aβ-induced inhibition of the OB network is concentration dependent and that this inhibition occurs at clinically relevant concentrations [53,54], which we believe do not necessarily cause neuronal loss. Previously, it was reported that Aβ oligomers can induce neuronal death [55,57], even at concentrations very close to those used in our experiments [55]. Here, we have exploited one of the advantages of the *in vitro* approach to show that acute application of Aβ induces a reduction of spontaneous network activity in OB slices that is “reversible”, which strongly suggests that it does not involve cell damage. Thus, it is very likely that Aβ-induced neuronal network dysfunction is produced by an alteration of specific cellular mechanisms involved in generating spontaneous network activity [89]. One such mechanism might be the well-known Aβ-induced reduction in synaptic transmission [45,51,52]. In the hippocampus, Aβ reduces both glutamatergic [45,51,52] and GABAergic synaptic transmission [51,90,91], which are known to be key elements for the generation of population activity in the OB [32,35,37,42,73,76,91-96]. Complex interactions among GABAergic inhibition provided by granular cells and interneurons [32,35,42,73,76,93-96] along with glutamatergic synapses both in the glomerulus and that provided by principal cells [35,42,73,76,96] are known to be essential for the generation of different patterns of activity in the OB [35,42,73,76,96]. Since Aβ disrupts both types of synaptic interactions in other neuronal networks [42,52] it is highly likely that Aβ-induced synaptic inhibition plays a major role in the alterations in OB network activity observed in this study. Interestingly, several studies in AD mouse models have suggested that a reduction in synaptic transmission occurs before any neuronal death and plaque formation and correlates with the neural dysfunction observed in these animals [15,55,97]. It remains to be determined to what extent the inhibition of OB network activity induced by acute application of Aβ to OB slices involves changes in the different synaptic interactions that occur in this network and whether these changes play a role in the long-term effects triggered by Aβ in the OB. It would be particularly interesting to test whether or not Aβ affects the dendrodendritic interaction between mitral and granular cells, which is a keystone of OB oscillatory capabilities [32,98-100].

Finally, we determined that a single application of Aβ in the OB affects olfaction two weeks after its injection (Figure 5), a phenomenon that is not accompanied by a change in motor performance or motivation to seek food (Figure 6). The observation that the advent of Aβ-induced smell loss occurs between the first and second week after its intrabulbar application strongly suggests that additional mechanisms, beyond the presence of the injected oligomers, are involved in this dysfunction (Figure 4). It is possible that injected Aβ oligomers trigger pathological events that need time to build up and that these secondary changes are responsible for sensory dysfunction. This finding is similar to previous observations that Aβ-induced impairment in learning and memory requires several days or weeks to develop [101-109]. Several processes can account for such delayed effects of Aβ microinjection. For instance, it is well known that Aβ inoculation can trigger central amyloidosis [102,105-117]. Accumulation and eventual precipitation of endogenous Aβ, such as that shown in Figure 4, can be achieved by the intracerebral inoculation of brain material containing Aβ seeds extracted from AD brains or from AD-transgenic mice [111-117] or by the microinjection of synthetic Aβ [102,105–110,116], but not of a scrambled Aβ sequence [102]. In both cases, these amylodogenic processes can take several weeks, or even months [117], to be established and are associated with a variety of pathological changes such as astroglial [105,106,108,109,112,113,115,118,119] and microglial reactions [105,109,112,113,118,119], appearance of dystrophic dendrites [112,113] neuroinflammation [111,118,119], endocrine stress [118,119], reduction in BDNF [118,119], tau phosphorylation [109], and reduction in synaptic transmission [102,105]. Whether our intrabulbar microinjection of Aβ is inducing one or several of these mechanisms and how they contribute to the induction of smell loss need to be determined. In any case, we found evidence of thiazine red positive deposits, probably Aβ accumulations [67-69], four weeks after the oligomers injection (Figure 4). It would be important to consider that long-term overproduction of Aβ has already been related to the neurodegeneration of olfactory sensory neurons [24], which raises the possibility that intrabulbar Aβ application would eventually induce some neuronal damage in the OB or its related networks. An alternative, or complementary, explanation for the delayed effects of intrabulbar application of Aβ on smell is that the pathological alterations produced by the injected Aβ may require the delayed transsynaptic modification of other olfactory networks beyond the olfactory bulb. This possibility is supported by the observations that injection of Aβ in a specific neural network, or its over-production, induces progressive transsynaptic alterations that gradually spread from the area of injection or over-production to brain regions with which it has prominent efferent connections [120,121]. Regardless of the mechanisms involved in the delayed Aβ-induced smell loss, our results support previous observations that transgenic animals expressing an AD-like phenotype have deficits in a variety of olfactory-related behaviors [12-20] that might involve alterations not only in the OB but also in other neural networks [13-19]. In fact, previous findings have linked early accumulation of soluble Aβ in the OB with olfactory dysfunction [15,20]. Since transgenic mice suffer a variety of adaptive changes that preclude establishing a direct, unequivocal relationship between the genetic alteration and the phenotypic outcome, the relationship between Aβ and olfactory dysfunction can be considered only a correlation [15,20]. Here, we show direct evidence that Aβ oligomers trigger a delayed loss of the ability to smell (Figure 4), which may be due to the actions of soluble and/or insoluble Aβ. Thus, our data support previous work showing that Aβ alters the activity of the OB network, resulting somewhat later in an inability to process olfactory information, as occurs in patients with AD and in transgenic animals [15,20,24]. Our demonstration of Aβ-induced OB dysfunction indicates that studies of the cellular mechanisms involved will help to understand AD pathology and reveal potential therapeutic targets against this disease.
